# Specific Antibodies Reacting with SV40 Large T Antigen Mimotopes in Serum Samples of Healthy Subjects

**DOI:** 10.1371/journal.pone.0145720

**Published:** 2016-01-05

**Authors:** Mauro Tognon, Alfredo Corallini, Marco Manfrini, Angelo Taronna, Janet S. Butel, Silvia Pietrobon, Lorenzo Trevisiol, Ilaria Bononi, Emanuela Vaccher, Giuseppe Barbanti-Brodano, Fernanda Martini, Elisa Mazzoni

**Affiliations:** 1 Sections of Pathology, Oncology and Experimental Biology, University of Ferrara, Ferrara, Italy; 2 Section of Microbiology, University of Ferrara, Ferrara, Italy; 3 Department of Molecular Virology and Microbiology, Baylor College of Medicine, Houston, Texas, United States of America; 4 Department of Surgery, University of Verona, Verona, Italy; 5 Department of Medical Oncology, Centro di Riferimento Oncologico, IRCCS, National Cancer Institute, Aviano, Italy; Second University of Naples, ITALY

## Abstract

Simian Virus 40, experimentally assayed *in vitro* in different animal and human cells and *in vivo* in rodents, was classified as a small DNA tumor virus. In previous studies, many groups identified Simian Virus 40 sequences in healthy individuals and cancer patients using PCR techniques, whereas others failed to detect the viral sequences in human specimens. These conflicting results prompted us to develop a novel indirect ELISA with synthetic peptides, mimicking Simian Virus 40 capsid viral protein antigens, named mimotopes. This immunologic assay allowed us to investigate the presence of serum antibodies against Simian Virus 40 and to verify whether Simian Virus 40 is circulating in humans. In this investigation two mimotopes from Simian Virus 40 large T antigen, the viral replication protein and oncoprotein, were employed to analyze for specific reactions to human sera antibodies. This indirect ELISA with synthetic peptides from Simian Virus 40 large T antigen was used to assay a new collection of serum samples from healthy subjects. This novel assay revealed that serum antibodies against Simian Virus 40 large T antigen mimotopes are detectable, at low titer, in healthy subjects aged from 18–65 years old. The overall prevalence of reactivity with the two Simian Virus 40 large T antigen peptides was 20%. This new ELISA with two mimotopes of the early viral regions is able to detect in a specific manner Simian Virus 40 large T antigen-antibody responses.

## Introduction

A new wave of investigations designed to assess the spread of Simian Virus 40 (SV40) infection in the human population was carried out over the last decade by different teams. Independent analyses reported the detection of SV40 DNA sequences and viral neutralizing and IgG antibodies in healthy subjects, including in children, adolescents, adults, elderly individuals, and pregnant women [1–6]. Similar investigations detected SV40 markers in patients affected by cancers of different histotypes [7–14].

SV40 is a monkey polyomavirus isolated originally in 1960 as a natural contaminant of early poliomyelitis vaccines, including both the Salk intramuscular inactivated vaccine and the Sabin oral attenuated vaccine [15]. Residual infectious SV40 survived in the inactivated Salk vaccines; higher levels of infectious SV40 were present in the live Sabin vaccine. These vaccines were administered world-wide to human populations [13,16]. Both inactivated and live attenuated vaccines had been produced in primary kidney cell cultures obtained from naturally infected wild Asian macaques (*Macacus rhesus*), which is the natural host for SV40. These poliovaccines accidentally contaminated with SV40 were employed during the period 1955–1963 [13,16–18]. However, it has been reported that SV40-contaminated anti-poliomyelitis vaccines were also administered in subsequent years [19]. The circulation of SV40 in human populations before the introduction of contaminated vaccines cannot be excluded.

Soon after its isolation, SV40 proved to be a viral transforming agent in different cultured animal and human cells and to be oncogenic in experimental animals [16,17,20,21]. SV40 transforming and oncogenic activities are mediated by the viral oncogene proteins, named large T (Tag) and small t (tag) antigens [20–23]. Tag is the major viral replication protein. Tag binds and abolishes functions of the tumor suppressor gene products of the p53 and pRb families, whereas tag enhances transformation by Tag by interacting with cellular protein phosphatase-2A. Altogether these data indicate that SV40 is a powerful oncogenic viral agent [16,17,20,21,24]. Human tumors of the same histotypes as those induced in animals have been examined for SV40 markers. Numerous studies have detected either SV40 sequences and/or the expression of its oncogene product, the Tag, in brain and bone tumors, mesothelioma and lymphoproliferative disorders [13,16,17].

Not all research groups reported the same epidemiologic data, indicating that this polyomavirus infection is present at different prevalences in distinct cohorts of human subjects [2,4,6,25–30]. An alternative hypothesis is that the different prevalence of SV40 infection could reflect different techniques employed in distinct investigations [16,31,32]. A recent proposal envisions that SV40 human infections resulted originally from exposure to contaminated oral poliovaccines; infections persist today (~50 years later) in populations and geographic regions where living conditions, poor sanitation, or unknown factors allow maintenance of infection, presumably transmitted by a fecal/urine–oral route [13,21]. This model predicts that SV40 prevalence rates will vary substantially, depending on the populations being studied.

Conflicting data showed the need for new standardized methods that could be shared among researchers involved in SV40 investigations [33]. We have recently contributed to this goal by developing an innovative indirect enzyme-linked immunosorbent assay (ELISA) with synthetic peptides, which mimic antigens of the viral capsid proteins 1, 2 and 3 (VPs 1–3). This indirect ELISA is specific for the detection of SV40 antibodies in human sera and does not react with different genetic variants of the closely related human polyomaviruses BKPyV and JCPyV or with other less-homologous human polyomaviruses [1–4,8,10–12,14]. Immunologic data obtained with this assay indicated that the prevalence of SV40 serum IgG antibodies in healthy adults is approximately 18%, whereas antibody prevalence is significantly higher (2–3 times) in patients affected by SV40-associated tumors [1–4,8,10–12]. Interestingly, the same indirect ELISA showed no association between SV40 and other human cancers, such as breast and undifferentiated nasopharyngeal carcinomas [27,28].

The detection of SV40 antibodies in human sera is an important parameter with which to measure the spread of viral infection in the human population and to verify the association of SV40 with human pathologies. We have developed an indirect ELISA with mimotopes representing the SV40 Tag oncoprotein. As this SV40 early gene product is critical for virus replication, transformation, and tumor activities, immunologic results with Tag mimotopes would provide key data for understanding the epidemiology of this small DNA tumor virus and its association with human tumors. In addition, such data could substantiate whether SV40 replicates in the human host and if SV40 is being transmitted among humans independently from exposure to contaminated vaccines.

## Materials and Methods

### Human samples

A new serum collection of 704 samples was harvested in 2014 from healthy subjects (HS), aged 18–65 years old, in the Clinical Laboratory of the University Hospital of Ferrara. These individuals were being seen for routine check-ups and Clinical Laboratory records indicated that they were in good health at the time. Sera were taken from discarded laboratory specimens after routine analyses were completed. Anonymously collected sera were coded with age and gender information only. The project was approved by the County Ethical Committee, Ferrara.

### In-silico structural analysis of Tag peptides A and D

Two SV40 large Tag linear peptides, designated peptide A (Tag A) and peptide D (Tag D), were characterized for stable secondary structure formation. Computer-assisted analysis was performed using programs of the PSIPRED web tool [34–36]. Peptide sequences were mapped on the native Tag protein to verify structural and functional similarities. SV40 Tag protein structure was obtained from computational predictions carried out by I-TASSER [37–39]. Molecular visualizations were performed by PyMOL (PyMOL Molecular Graphics System, Version 1.3, Schrödinger, LLC). Computational tools were available through the ExPASy server [40].

### Synthetic peptides

**Fig 1:**
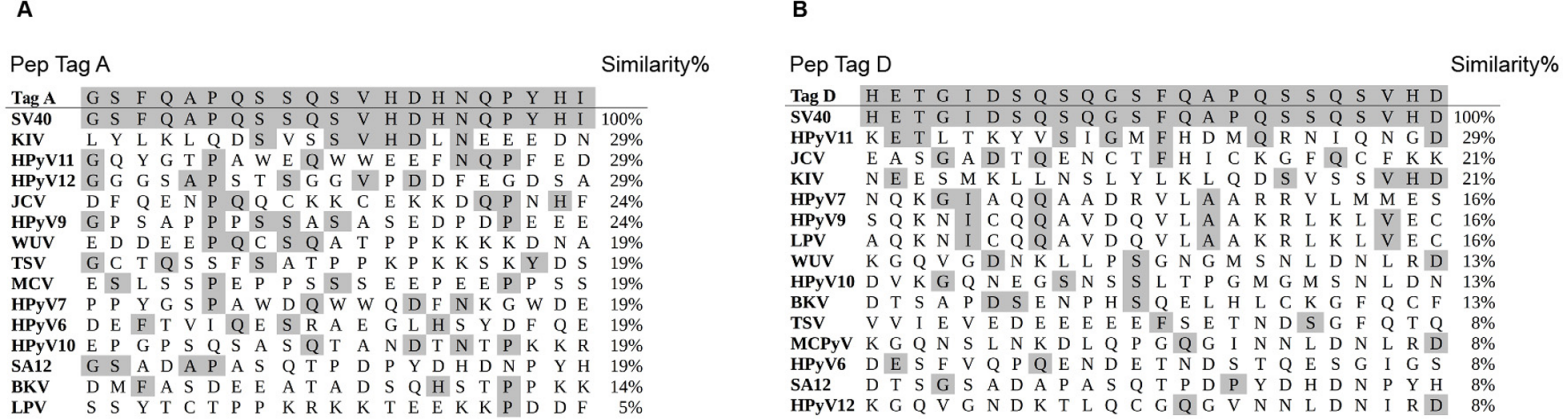
Similarity among SV40-specific Tag mimotopes (Pep Tag A and Pep Tag D) and other polyomavirus Tag sequences, Panel A and B, respectively. SV40 (simian virus 40) Tag sequences were compared to the Tags of the following polyomaviruses: BKV (BKPyV, BK polyomavirus), HPyV6 (human polyomavirus 6), HPyV7 (human polyomavirus 7), HPyV9 (human polyomavirus 9), HPyV10 (human polyomavirus 10), HPyV11/STLPyV (human polyomavirus 11), HPyV12 (human polyomavirus 12), JCV (JCPyV, JC polyomavirus), KIV (KIPyV, KI polyomavirus), LPV/AGMPyV (B-lymphotropic polyomavirus), MCV (MCPyV, Merkel cell polyomavirus), SA12 (simian agent virus 12), TSV (TSPyV, Trichodysplasia spinulosa-associated polyomavirus), WUV (WUPyV, WU polyomavirus), and NJPyV (New Jersey polyomavirus, not shown). (http://www.ncbi.nlm.nih.gov/Taxonomy/Browser/wwwtax.cgi?id=10624).

**Fig 2:**
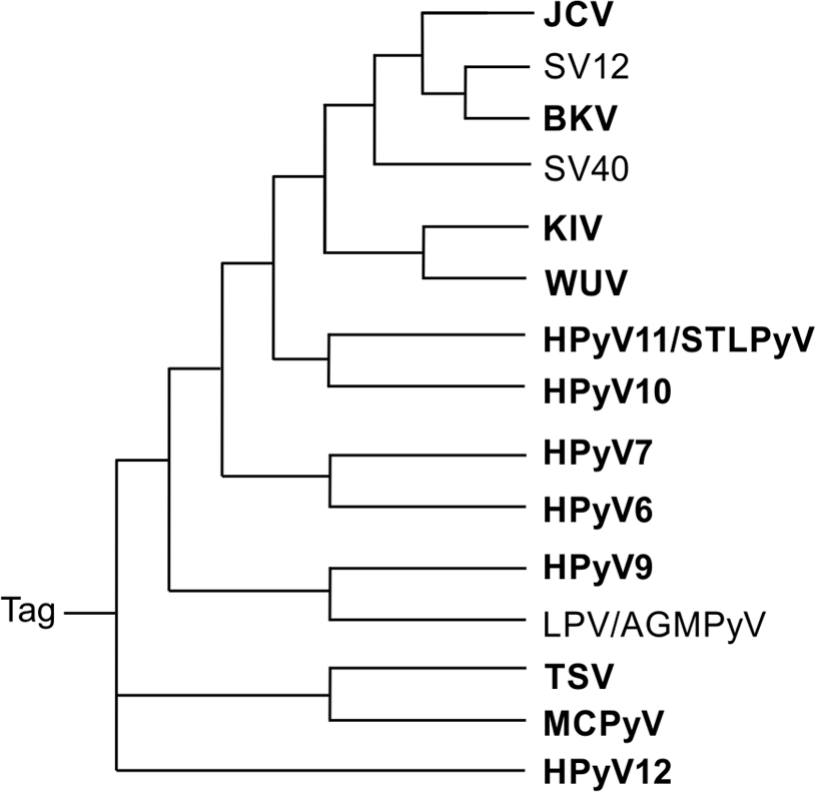
Polyomavirus phylogenetic tree based on the large Tag a.a. sequences. The similarity of large Tag sequences among different polyomaviruses is shown. Note that SV40 (simian virus 40) Tag is more closely related to those of JCV (JCPyV, JC polyomavirus), SA12 (simian agent virus 12), and BKV (BKPyV, BK polyomavirus) than to the Tags of other polyomaviruses: KIV (KIPyV, KI polyomavirus), WUV (WUPyV, WU polyomavirus), HPyV11/STLPyV (human polyomavirus 11), HPyV10 (human polyomavirus 10), HPyV7 (human polyomavirus 7), HPyV6 (human polyomavirus 6), HPyV9 (human polyomavirus 9), LPV/AGMPyV (B-lymphotropic polyomavirus), TSV (TSPyV, Trichodysplasia spinulosa-associated polyomavirus), MCPyV (Merkel cell polyomavirus), HPyV12 (human polyomavirus 12), and NJPyV (New Jersey polyomavirus, not shown) (http://www.ncbi.nlm.nih.gov/Taxonomy/Browser/wwwtax.cgi?id510624).

**Fig 3:**
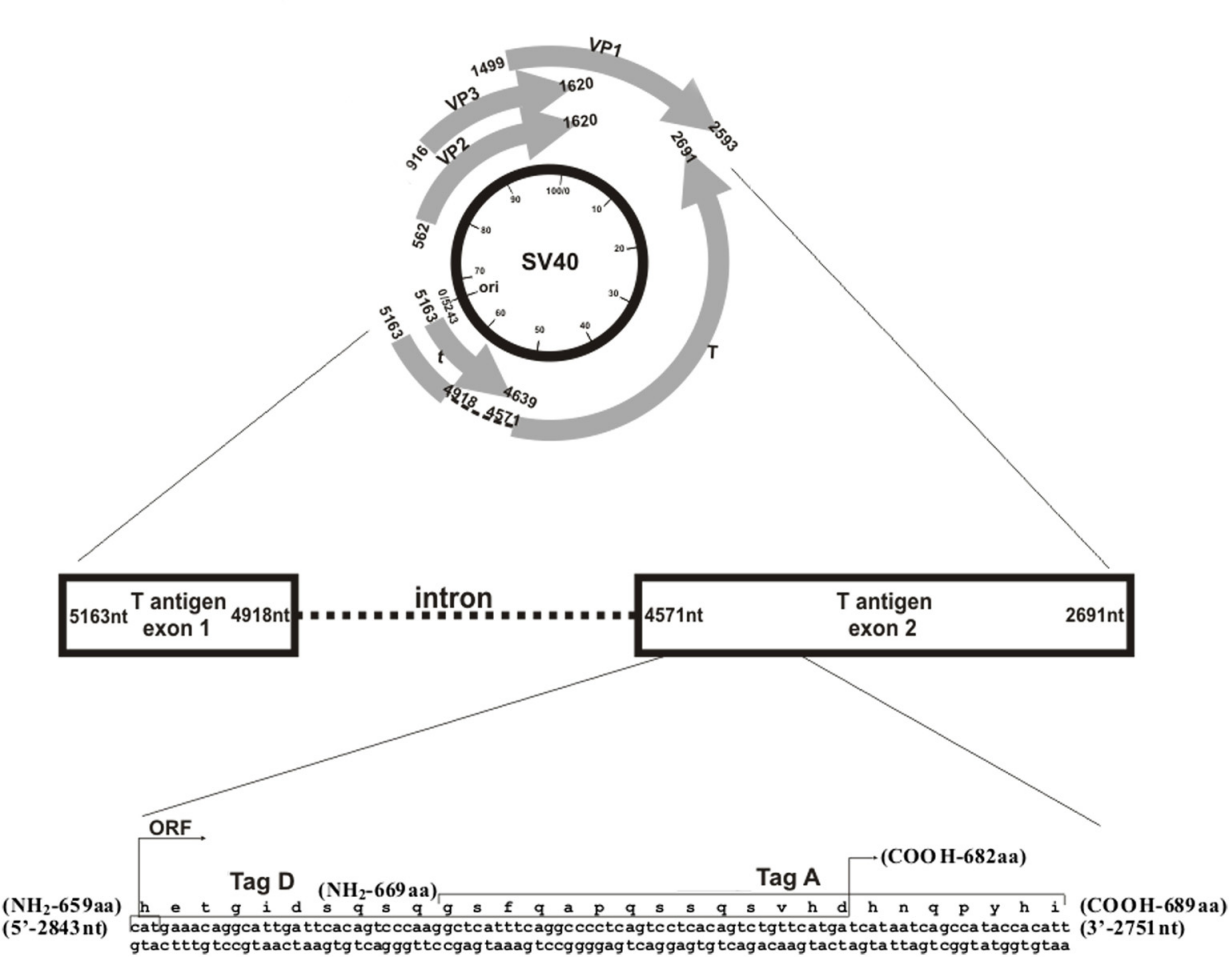
Simian virus 40 (SV40) genome and the two selected peptides from the early coding region employed in indirect ELISA. The upper panel represents a circular map of the SV40 genome with map units from 0 to 100 running in a clockwise direction (inner circle, black). The nucleotide (nt) sequence and numbers refer to the 5,243 nucleotide genome of SV40 strain 776 (http://www.ncbi.nlm.nih.gov/genome). Ori marks the origin of viral DNA replication (0/5243 nt). SV40 early and late genes are transcribed in anti-clockwise and clockwise directions, respectively (gray arrows); numbers indicate nt. The large T antigen (T) and small t antigen (t) are encoded by the early region. T antigen exon 1 is encoded from 5,163–4,918 nt and exon 2 from 4,571–2,691 nt, with intron 1 from 4,917 to 4,572 nt. Small t antigen is encoded from 5,163 to 4,639 nt. Viral capsid proteins 1–3 are codified by the late region (VP1, 1,499–2,593 nt; VP2, 562–1,620 nt; and VP3, 916–1,620 nt). A portion of the early coding region is expanded at the bottom of the figure. The selected Tag peptides, Tag A and Tag D, are from exon 2; Tag A encompasses amino acids (a.a.) 669–689 (21 a.a.), and Tag D a.a. 659–682 (24 a.a.). These two Tag epitopes overlap from a.a. 669 to 682.

Computer-assisted analyses enabled us to select the 2 specific SV40 Tag peptides A and D (Fig 1) from the viral early region. The selection was carried out as described previously for SV40 VP peptides [2,12]. Briefly, SV40 Tag mimotopes were compared with Tag amino acids (a.a.) from human polyomaviruses BKPyV and JCPyV, which are highly homologous to SV40, as well as with other, less homologous, polyomaviruses (web site, http://blast.ncbi.nlm.nih.gov) (Fig 2). The amino acid residues of the two specific SV40 Tag peptides showed low homology with the T-antigens of BKPyV and JCPyV (Fig 1). The location of the two peptides A and D in exon 2 of the Tag oncoprotein is shown (web site, http://www.ncbi.nlm.nih.gov/nuccore) (Fig 3). The a.a. sequences of the two peptides Tag A and Tag D are as follows: (a) Tag A: NH2-G S F Q A P Q S S Q S V H D H N Q P Y H I-COOH (Tag a.a. 669–689), and (b) Tag D: NH2-H E T G I D S Q S Q G S F Q A P Q S S Q S V H D-COOH (Tag a.a. 659–682). The two peptides overlap from Tag a.a. 669 to 682. SV40 Tag A and Tag D mimotopes were selected as they reacted specifically in indirect ELISA (see below) testing with a rabbit hyperimmune serum prepared against purified SV40 Tag protein (positive control sera). BKPyV and JCPyV hyperimmune sera did not react with SV40 Tag A and Tag D peptides (negative control sera). The human neuropeptide S (hNPS), a.a. sequence SFRNGVGTGMKKTSFQRAKS, which is SV40 unrelated, was employed as a human negative control peptide [41]. The peptides were synthesized by standard procedures and purchased from UFPeptides s.r.l., Ferrara, Italy.

### Hyperimmune rabbit and human control sera

Hyperimmune serum against SV40 Tag was obtained from rabbits, which had been immunized with purified Tag protein. Briefly, SV40 Tag was immunoprecipitated from the SV40-transformed mouse cell line MKS-A using the mouse monoclonal antibody against Tag, Pab 430, as described [42]. The mKS-A cell line had been established by SV40 transformation of primary BALB/c mouse kidney cells [43] and obtained from LW Law [44]. After immunoprecipitation, Tag was purified by SDS-polyacrylamide gel electrophoresis and gel slices were recovered, processed, and mixed with Freund’s adjuvant for injection. New Zealand white rabbits were injected intramuscularly in the hind flank with 0.5 ml of Tag/adjuvant mixture. The first Tag protein injection used complete Freund’s adjuvant, whereas subsequent injections at 4-week intervals used incomplete Freund’s adjuvant. Approximately 70 μg of Tag protein diluted in 500 μl of emulsified adjuvant was employed for each injection. A total of six injections were given. Bleeds were taken during the immunization period and 15 days after the last Tag immunization. The sera were tested by immunofluorescence (IF), immunoprecipitation (IP), and Western blot (WB) analysis. A high-titer pool of hyperimmune rabbit serum (IF titer = 1/500; WB titer = 1/500; IP = 10 μl undiluted) was employed in the experiments described here. Rabbit experiments were performed in compliance with relevant laws and institutional guidelines and in accordance with the ethical standards of the Declaration of Helsinki. The experiments in rabbits were approved by the Baylor College of Medicine Institutional Animal Care and Use Committee (https://www.bcm.edu/gs/LinkedPowerpoints/Ethics-2L2-Laboratory%20Animals.pdf).

Six human sera found to be SV40 VP positive with neutralizing activities in a previous study [8] were employed as additional positive controls in all indirect ELISA carried out with SV40 Tag mimotopes A and D. They gave optical density readings of 0.25–0.72.

### Indirect ELISA

An indirect ELISA was developed to detect specific antibodies against SV40 Tag in human sera. ELISA plates were coated with synthetic peptides, which were employed as mimotopes, corresponding to SV40 large Tag encoded by the early viral DNA region. Synthetic peptides, Tag A (21 a.a.) and Tag D (24 a.a.), were described above.

#### Peptide coating

96 flat-bottom well plates (Nunc-immuno plate PolySorp, CelBio, Milan) were coated with 5 μg of the selected peptide in each well, diluted in 100 μl of Coating Buffer, pH 9.6 (Candor Bioscience, Weissensberg, Germany). The plates were incubated at 4°C for 16 hours, allowing the peptide to cover the bottom of each well completely. Plates were rinsed three times with Washing Buffer (Candor Bioscience, Germany) to remove unattached peptide.

#### Peptide blocking

Blocking was accomplished with 200 μl/well of Blocking Solution containing casein (Candor Bioscience, Germany) at 37°C for 90 min to allow for well saturation.

#### Primary antibody

To eliminate residual blocking solution, the plates were rinsed three times with Washing Buffer using a washing apparatus (Thermo Electron Corp., model Wellwash 4MK2, Finland). Different wells then were covered with 100 μl containing the following sera: positive control represented by immune rabbit serum containing anti-SV40 Tag antibodies; negative controls represented by immune sera with anti-BKPyV and anti-JCPyV antibodies and three human serum samples found to be SV40 negative in previous investigations. Test human serum samples from HS were diluted in Low Cross-Buffer pH 7.2 (Candor Bioscience, Germany) at 1:20 initially (and subsequently at dilutions up to 1:160) and were added to the plate. Additional controls in each plate included a well with the secondary antibody only and other wells void of both primary and secondary antibodies. The plate was incubated at 37°C for 90 min.

#### Secondary antibody

After 90 min of incubation, a triple rinsing cycle was repeated and then the secondary antibody solution was added to each well. The solution contained goat anti-human or goat anti-rabbit IgG heavy (H) and light (L) chain specific peroxidase-conjugate (Calbiochem-Merck, Germany) diluted 1:10,000 in Low Cross-Buffer. The reaction mixture was incubated at room temperature for 90 min.

#### Optical density reading

At the end of the incubation period, the plates were rinsed three times with Washing Buffer and then treated with 100 μl of 2,2'-azino-bis 3-ethylbenzthiazoline-6-sulfonic acid (ABTS) solution (Sigma-Aldrich, Milan) which reacted with the peroxidase enzyme to yield the color reaction. The plate was then read spectrophotometrically (Thermo Electron Corp., model Multiskan EX, Finland) at a wavelength (λ) of 405 nm. This optical density (OD) reading reflected the extent of immunocomplexes formed by the presence of specific antibodies which bound to the SV40 synthetic peptide/epitopes/mimotopes.

The three SV40 negative control sera were selected from below the cut-off value determined with second–degree polynomial regression by plotting the ranked net OD individual values for each peptide. A tendency curve was drawn from a second–degree polynomial regression for Tag A peptide and Tag D peptide, as published for MCPyV and BKPyV virus-like particles (VLPs) [45]. Our representations revealed an inflection point corresponding to 0.18 for each peptide.

#### Cutoff

Cut-off values were determined for each assay by the OD reading of the three negative control sera that were added to the standard deviation and multiplied three times (+3SD). Sera with antibodies against SV40 were considered Tag-positive if they reacted to both Tag peptides all three times in three independent ELISA tests.

### Statistical analysis

Statistical analyses were performed by Prism 4.0 (GraphPad software). To determine significances among HS groups, the Chi-square test with Yates’ correction was used. The serologic profile of serum antibody reactivity to SV40 mimotopes was statistically analyzed using Anova and Newman-Keuls Comparison test. *P* values ≤ 0.05 were considered to be statistically significant.

## Results

### Computational analysis of SV40 Tag peptides

**Fig 4:**
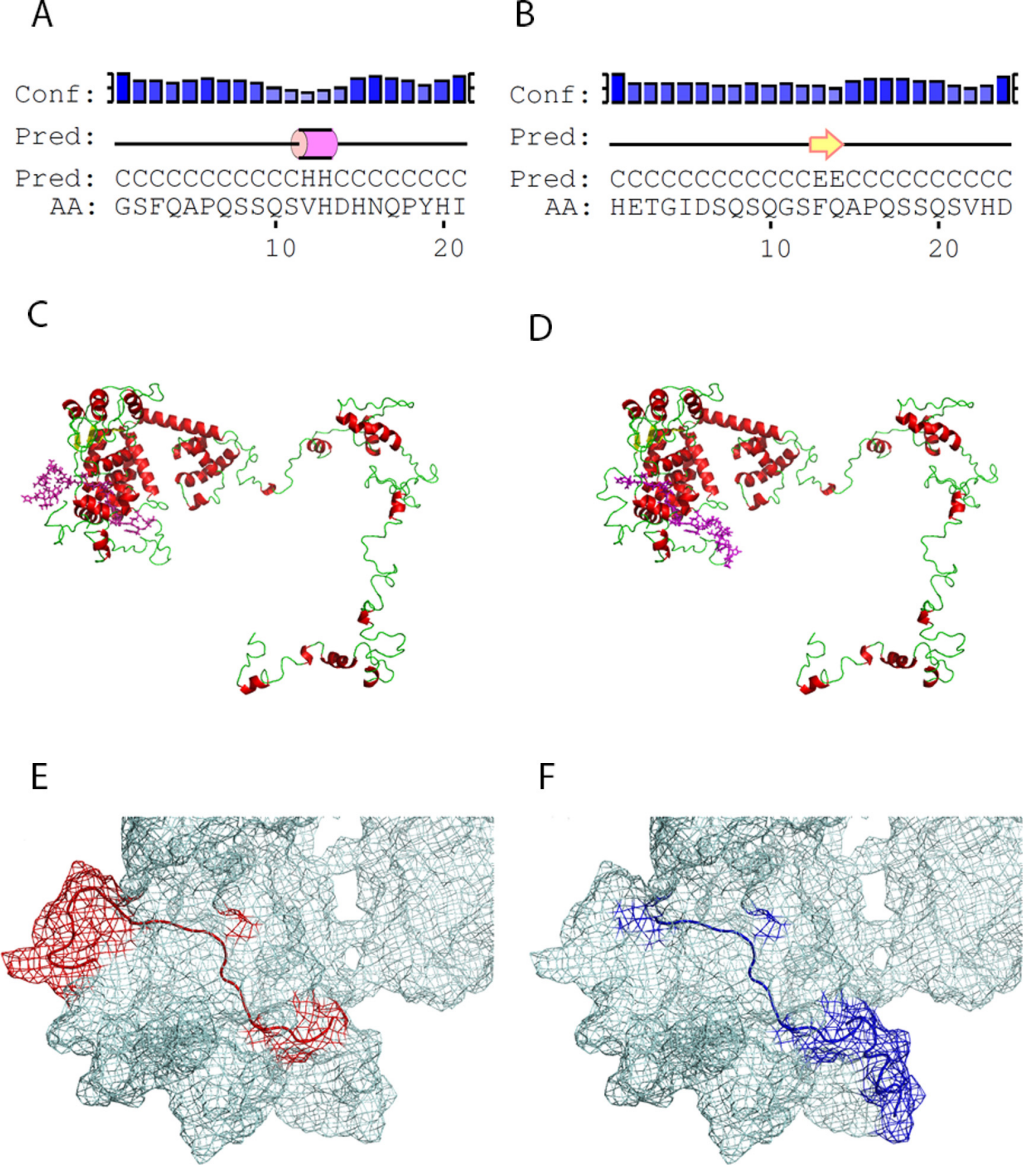
Structural characteristics of large T-antigen peptides. (A) Tag A and (B) Tag D linear peptides were characterized by secondary structure folding domains identified by PSIPRED analysis. (C and D) Tertiary structures of SV40 large T antigen computationally determined with I-TASSER modeling algorithm, with a C-score = –2. Tag A and D both show a partial overlapping localization near the N-terminal native protein domain (magenta sticks in panel C and D, respectively). (E and F) Mesh representation of the large T antigen surface (N-terminal portion) on which Tag A and Tag D are mapped.

Computational analysis showed that the linear Tag peptides are characterized by secondary structure folding domains identified as follows (PSIPRED). Tag A contains a small alpha helix domain involving a.a. 12 and a.a. 13. Tag D forms a beta strand secondary structure containing two amino acid residues, a.a. 13 and a.a. 22 (Fig 4). The structures obtained had the highest statistical confidence rates for each residue. Overall, the secondary structure prediction methods employed achieved an average accuracy of 75%. Tertiary structures of SV40 large Tag were selected among the computationally determined predictions. For estimating the accuracy of the three-dimensional structure predictions, a confidence score (C-score) was defined based on the quality of the threading alignments and the convergence of the I-TASSER’s structural assembly refinement simulations. C-score is typically in the range [−5, 2], wherein a higher score reflects a model of better quality. The Tag model selected presented a C-score = –2. Mapping of the linear peptides on the inferred large Tag protein structures indicated that Tag A and Tag D both show a random coil conformation with partial exposition of the a.a. chain on the surface of the large Tag molecule (Fig 4).

### Detection of SV40 Tag antibodies by indirect ELISA

**Fig 5:**
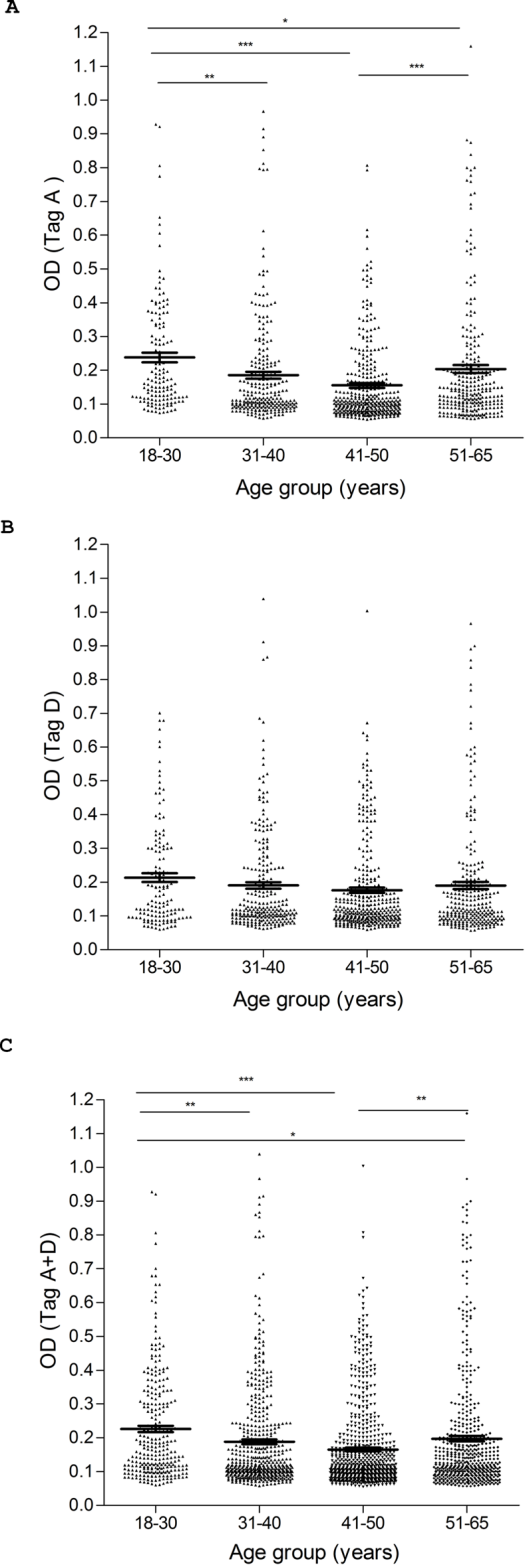
Serologic profile of human serum antibody reactivity to SV40 large T antigen mimotopes Tag A (A), Tag D (B) and Tag A+D (C). Immunologic data are from healthy subjects (HS). Results are presented as values of OD readings at λ 405 nm for serum samples diluted 1:20 and assayed in indirect ELISA. In this scatter dot plotting, each plot represents the dispersion of individual sample OD values to a mean level, indicated by the long horizontal line inside the scatter with standard error of the mean (SEM) marked by short horizontal lines for each age group. The OD readings of serum samples were stratified by age: 18–30 years (yrs), 31–40 yrs, 41–50 yrs, and 51–65 yrs. Data were analyzed with one way Anova analysis and Newman-Keuls Multiple Comparison test (OD mean, 95% CI). (A) High levels of antibodies against SV40 Tag A in HS aged 18–30 yrs (0.24 OD, 95% CI = 0.21–0.27) vs. HS aged 31–40 yrs (0.18 OD, 95% CI = 0.16–0.20, **P < 0.01), vs. HS aged 41–50 yrs (0.15 OD, 95% CI = 0.14–0.17, ***P < 0.001), vs HS aged 51–65 (0.20 OD, 95% CI = 0.18–0.23, *P<0.05). High levels of antibodies against HS aged 51–65 yrs vs HS aged 41–50 yrs (***P<0.001). (B) Levels of antibodies against SV40 Tag D were not statistically different among age groups of HS (P > 0.01). (C) High levels of antibodies against both SV40 Tag peptides Tag A+D were observed in HS aged 18–30 yrs (0.23 OD, 95% CI = 0.21–0.24) vs. HS aged 31–40 yrs (0.19 OD, 95% CI = 0.17–0.20, **P < 0.01), vs. HS aged 41–50 yrs (0.16 OD, 95% CI = 0.15–0.18, ***P < 0.001), and vs HS aged 51–65 yrs (0.20 OD, 95% CI = 0.18–0.21, *P<0.05). High levels of antibodies against HS aged 51–65 yrs vs HS aged 41–50 yrs (**P<0.01).

**Fig 6:**
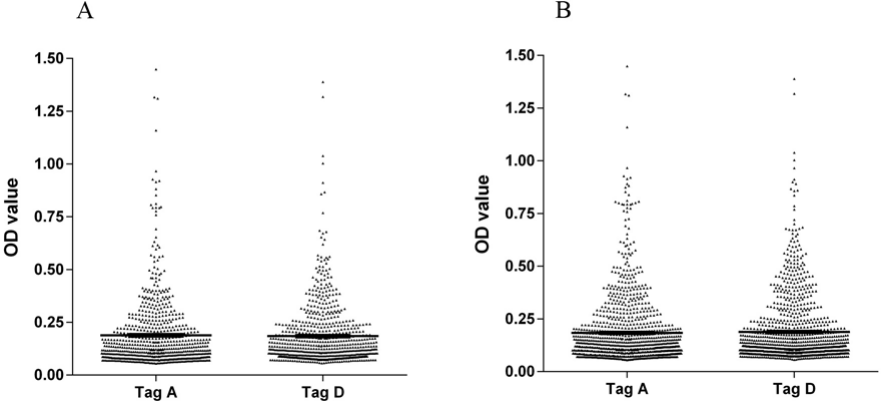
Intra-run and inter-run variability of OD values of human serum antibody reactivity to Tag A and D peptides. Data are presented as scatter dot plot of OD readings at λ 405 nm, mean and standard error of the mean (SEM) marked by short horizontal lines for each peptide. (A) OD value variability, intra-run. Tag A: mean = 0.19, SEM = 0.006; Tag D: mean = 0.18; SEM = 0.005. (B) OD value variability, inter-run. Tag A: mean = 0.18, SEM = 0.005; Tag D: mean = 0.19, SEM = 0.005.

The aim of this investigation was to determine the presence of serum antibodies against SV40 Tag in HS. Human sera, from HS aged 18–65 yrs, were analyzed for IgG antibodies which could react with SV40 Tag peptides. This immunologic analysis employed the novel indirect ELISA described in Materials and Methods. Synthetic peptides corresponding to SV40 Tag epitopes, named A and D mimotopes, together with an SV40-unrelated human peptide hNPS as a negative control, were used. Rabbit anti-Tag positive antiserum prepared against purified SV40 Tag reacted with Tag A and Tag D mimotopes with OD readings of 2.9 and 2.2, respectively. This confirmed that the two Tag mimotopes were immunogenic. Human serum samples were tested initially at a dilution of 1/20 for reactivity to SV40 Tag A peptide (Table 1). Samples reactive with the Tag A mimotope (166 of 704 tested) reached an overall prevalence of 24%, with the OD for SV40 Tag-positive samples ranging from 0.18 to 1.45 (Fig 5). When the serum samples were tested against the second SV40 Tag-specific synthetic peptide, the Tag D mimotope, samples reacted with the Tag D peptide (169 of 704 tested) with the same prevalence of 24% as observed for the Tag A peptide, with the OD ranging from 0.18 to 1.38. Serologic profiles of serum antibody reactivity to SV40 mimotopes are presented in Fig 5, whereas intra-run and inter-run variability is illustrated in Fig 6.

**Table 1:** Prevalence of immunoglobulin G antibodies in sera from healthy subjects reactive with simian virus 40 (SV40) large T-antigen mimotopes.

Age year	Number of Subject	Number of Positive Samples (%) Peptides tested^a^			
		Tag A	Tag D	Tag (A+D)	hNPS
18–30	88	32 (36)	28 (32)	25 (28)*	0 (0)
31–40	171	38 (22)	49 (29)	35 (20)	0 (0)
41–50	255	60 (24)	61 (24)	49 (19)	0 (0)
51–65	190	36 (19)	31 (16)	29 (15)*	0 (0)
Total	704	166 (24)	169 (24)	138 (20)	0 (0)

Human sera were from healthy subjects. Statistical analysis was performed using the Chi-square test with Yates’ correction. The difference in prevalence of SV40 antibodies between the cohorts of individuals aged 18–30 years old and 51–65 years old was statistically significant (*P < 0.05).

^a^Tag A = SV40 T antigen mimotope A; Tag D = SV40 T antigen mimotope D

hNPS = human neuropeptide S.

The human negative control peptide hNPS did not react with any of the sera tested (Table 1). The OD value was usually in the range of 0.050–0.080, consistent with the OD of SV40-negative sera. Human sera that tested positive for both SV40 Tag mimotopes A and D totaled 138, for an overall prevalence of 20% (Table 1). These sera were considered to be SV40 Tag positive. The majority of serum samples found to be negative for the Tag A peptide (n = 507/704; 72%) also failed to react with SV40 Tag D antigen. Some serum samples (n = 59/704; 8%) were found to be discordant; n = 28 were reactive for the Tag A peptide but tested negative for the Tag D peptide, whereas n = 31 sera reacted with the Tag D peptide but were Tag A negative. Sera that reacted with only one Tag peptide were not counted as positive. The difference in prevalence of reactivity for the Tag A (166/704; 24%) and Tag D (169/704; 24%) peptides was not statistically significant (*p*>0.05). Similarly, the difference in seroprevalence for either peptide A or peptide D (24%) and for peptides A+D (20%) was not statistically significant.

In addition to the Tag-positive rabbit serum control, six SV40 Tag-positive human control sera, which reacted with both peptides A and D, were selected from sera which had reacted with SV40 VP mimotopes in a previous investigation. The SV40 Tag-positive human sera displayed ODs up to 1.4, whereas human sera that did not react with SV40 Tag mimotopes had low ODs of less than 0.18. Additional controls were 9 BKPyV and 10 JCPyV positive human sera with high OD readings against those human polyomaviruses from patients affected with neurological diseases, including multiple sclerosis, which were analyzed in a previous study [46]. Herein, those sera tested negative for SV40 Tag (OD <0.18). The lack of cross-reactivity by those sera further supports the specificity of our indirect ELISA for SV40 Tag.

Small differences were detected in age-related prevalence of serological SV40 Tag-positivity (Table 1). In our investigation only those samples found positive for both peptides A and D were considered SV40-positive. Prevalences of 28%, 20%, 19%, and 15% were observed in subjects in age groups 18–30, 31–40, 41–50, and 51–65 years old, respectively. These data suggest that there may be a gradual decline in SV40 Tag seropositivity with advancing age. However, that decline was statistically significant only when comparing the youngest [aged 18–30 (28%)] and the oldest [aged 51–65 (15%)] groups (*P* < 0.05) (Table 1).

To determine Tag antibody titers, 20 HS sera found to be SV40-positive for both Tag A and Tag D peptides were serially diluted from 1/20 to 1/160 and investigated by indirect ELISA against both mimotopes. Antibodies against SV40 Tag remained detectable at a dilution of 1/160 in many sera (10/20, 50%). The titers of SV40 Tag antibodies in positive sera did not differ greatly against the two different Tag-specific peptides. The reproducibility of the serological results was assessed in three replicate experiments carried out by independent operators with no data variability.

## Discussion

The SV40-encoded large Tag is an essential protein required for viral replication, transformation, and oncogenicity [20,21,24]. Tag expression has been detected in human brain tumors, mesotheliomas, and different lymphoproliferative disorders, including AIDS-related lymphomas [13,14,16,17,20,29,47–51]. We describe here an indirect ELISA based on peptides representing epitopes of the SV40 Tag.

Bioinformatics analysis of Tag peptides A and D revealed secondary structures similar to those detected in the native Tag protein. Although the linear peptides contain small secondary structural domains (an alpha helix in Tag A and a beta strand in Tag D involving two a.a. residues each), their secondary structures are predominantly linear. Both Tag peptides contain domains exposed on their surfaces similar to those identified in the native Tag protein. These results suggest that the linear Tag peptides A and D resemble natural SV40 Tag epitopes that could be recognized by serum antibodies due to the peptide secondary and tertiary structures acting as docking sites.

An indirect ELISA was then developed using these two synthetic peptides to analyze serum samples from HS for reactivity to SV40 Tag. Detection of IgG antibodies against Tag in human sera suggest that SV40 infections had occurred in approximately 20% of the healthy adults surveyed. This antibody prevalence is relatively low, similar to what has been detected against SV40 capsid antigens using VP epitope peptides [1–4].

SV40 Tag antibody prevalence in human sera described here does not differ substantially from the SV40 seroprevalence for certain subgroups of subjects reported in earlier studies using the plaque reduction neutralization test against SV40 infectivity [1,2,6,52,53]. That assay is considered the gold standard for measuring the presence of SV40 antibody with neutralization activity. Some groups of subjects displayed lower rates of SV40 antibody, suggesting that SV40 infections are not ubiquitous. Similar findings of SV40 footprints were revealed by PCR assays in PBMC/buffy coat samples from normal individuals [52,54].

Earlier results with enzyme immunoassays were obtained mainly with recombinant VLPs or VP1 as antigens, which contain many common epitopes among SV40, BKPyV and JCPyV [55]. The sera were pre-absorbed with BKPyV and JCPyV antigens in an attempt to give SV40 specificity to the assay [30,55]. This technical procedure may have eliminated or drastically reduced the presence of SV40-specific antibodies in the sera [31].

SV40 sequences have been detected in different human tissues/samples [16,17,21,56]. These data are compatible with observations on subjects administered SV40-contaminated vaccines by different routes. In those vaccinèes, SV40 was detected and isolated from stools or throats up to several weeks following oral or nasal spray administration of contaminated vaccines. Under experimental conditions, SV40 is able to infect and multiply in both B and T lymphocytes, producing viral progeny at low titers [57].

We observed here an age-dependent prevalence of SV40 Tag antibody in HS, ranging from 28% positivity in donors 18–30 years old to 15% in HS aged 51–65 years old (Table 1). The difference between those two groups was statistically significant (*p* < 0.05). The reduced prevalence of SV40-positivity in older individuals was previously noted and could be ascribed to an age-dependent decline of immune surveillance [25].

In summary, we report here a novel indirect ELISA that employs specific SV40 peptides from the large Tag protein sequence for the detection of SV40 Tag antibodies in human sera. This is the first study to report overlapping immunologic reactivity in human sera for two SV40 Tag antigens/mimotopes (A+D). The SV40 Tag ELISA, as previously reported for the SV40 VP ELISA, gives reliable results which can be obtained from many samples in a short period of time at affordable costs. This Tag ELISA may provide the scientific community with a standardized assay applicable to studies of SV40 infection in human populations and of viral association with human cancers. Immunological data from this study suggest that SV40 is causing human infections. Alternatively, it is possible that our serologic findings could be due to infections by a closely related, currently undiscovered, human polyomavirus.
